# Machine Learning Model to Identify Sepsis Patients in the Emergency Department: Algorithm Development and Validation

**DOI:** 10.3390/jpm11111055

**Published:** 2021-10-21

**Authors:** Pei-Chen Lin, Kuo-Tai Chen, Huan-Chieh Chen, Md. Mohaimenul Islam, Ming-Chin Lin

**Affiliations:** 1 Graduate Institute of Biomedical Informatics, College of Medicine Science and Technology, Taipei Medical University, Taipei 106, Taiwan; peichen@berkeley.edu (P.-C.L.); d610106004@tmu.edu.tw (M.M.I.); 2 Emergency Department, Taoyuan General Hospital, Ministry of Health and Welfare, Taoyuan 330, Taiwan; 3 Emergency Department, Chi-Mei Medical Center, Tainan 710, Taiwan; 890502@mail.chimei.org.tw; 4 Department of Neurosurgery, Taipei Medical University-Wan Fang Hospital, Taipei 116, Taiwan; dissector@gmail.com; 5 Taipei Neuroscience Institute, Taipei Medical University, Taipei 110, Taiwan; 6 International Center for Health Information Technology (ICHIT), Taipei Medical University, Taipei 110, Taiwan; 7 Research Center of Big Data and Meta-Analysis, Wan Fang Hospital, Taipei Medical University, Taipei 116, Taiwan; 8 Department of Neurosurgery, Shuang Ho Hospital, Taipei Medical University, New Taipei City 235, Taiwan

**Keywords:** sepsis, septic shock, machine learning, emergency department, intensive care unit

## Abstract

Accurate stratification of sepsis can effectively guide the triage of patient care and shared decision making in the emergency department (ED). However, previous research on sepsis identification models focused mainly on ICU patients, and discrepancies in model performance between the development and external validation datasets are rarely evaluated. The aim of our study was to develop and externally validate a machine learning model to stratify sepsis patients in the ED. We retrospectively collected clinical data from two geographically separate institutes that provided a different level of care at different time periods. The Sepsis-3 criteria were used as the reference standard in both datasets for identifying true sepsis cases. An eXtreme Gradient Boosting (XGBoost) algorithm was developed to stratify sepsis patients and the performance of the model was compared with traditional clinical sepsis tools; quick Sequential Organ Failure Assessment (qSOFA) and Systemic Inflammatory Response Syndrome (SIRS). There were 8296 patients (1752 (21%) being septic) in the development and 1744 patients (506 (29%) being septic) in the external validation datasets. The mortality of septic patients in the development and validation datasets was 13.5% and 17%, respectively. In the internal validation, XGBoost achieved an area under the receiver operating characteristic curve (AUROC) of 0.86, exceeding SIRS (0.68) and qSOFA (0.56). The performance of XGBoost deteriorated in the external validation (the AUROC of XGBoost, SIRS and qSOFA was 0.75, 0.57 and 0.66, respectively). Heterogeneity in patient characteristics, such as sepsis prevalence, severity, age, comorbidity and infection focus, could reduce model performance. Our model showed good discriminative capabilities for the identification of sepsis patients and outperformed the existing sepsis identification tools. Implementation of the ML model in the ED can facilitate timely sepsis identification and treatment. However, dataset discrepancies should be carefully evaluated before implementing the ML approach in clinical practice. This finding reinforces the necessity for future studies to perform external validation to ensure the generalisability of any developed ML approaches.

## 1. Introduction

Sepsis, defined as “life threatening organ dysfunction caused by a dysregulated host response to infection” [1], is a global health problem with high mortality and morbidity [2]. Epidemiologic estimates have reported that the crude mortality of sepsis patients is over 20% [2,3,4,5], and the global cost of sepsis is estimated to be $16.7 billion [6,7]. Numerous studies [8,9,10] have demonstrated that timely identification of sepsis and initiation of an evidenced-based treatment protocol could decrease in-hospital mortality, shorten length of stay and reduce healthcare costs. Nevertheless, because of the heterogeneity of infectious insults and the diversity of hosts, efficiently recognising and treating sepsis remains highly challenging for physicians [11].

Early sepsis identification relies upon clinical data that is readily available during hospitalisation [12]. The currently available clinical sepsis risk scores, namely the Systemic Inflammatory Response Syndrome (SIRS) and quick Sequential Organ Failure Assessment (qSOFA), have several shortcomings, which hamper their utilisation in identifying the early signs of organ failure [13,14,15]. Therefore, it is urgently needed to develop a more precise and personalised tool to recognise sepsis in a timely manner. The increasing availability of electronic health records (EHR) and advancing machine learning (ML) techniques has stimulated attempts to identify patient conditions through the automated analysis of medical records. Previous studies have shown that the ML approach can facilitate the detection of sepsis and septic shock [10,16,17,18]. However, the clinical utility of these models in the emergency department (ED) setting remains uncertain. The majority of previous studies developed and validated the ML models using clinical data only from the intensive care unit (ICU) [18,19,20,21,22,23].

To better evaluate the clinical utility of ML approaches for identifying sepsis patients in the ED, we developed an ML technique to correctly identify sepsis patients, using clinical predictors available in the electronic health record (EHR). Afterward, we validated our model externally using a distinct dataset from a geographically separate institute that provided a different level of care. Finally, we compared model performance with currently available risk scores.

## 2. Materials and Methods

**Figure 1:**
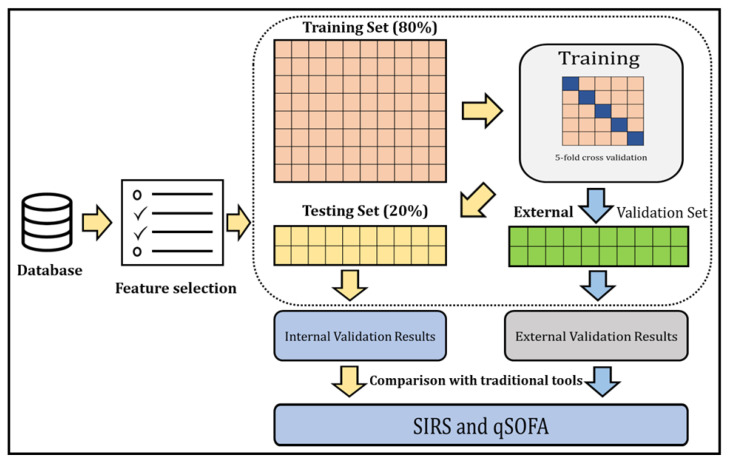
A general outline of the step-by-step approach to identify sepsis patients and compare the identification with traditional tools.

Overview of the study: The ML model was developed and externally validated to identify sepsis patients in the ED. The overall process of our study is depicted in Figure 1.

### 2.1. Study Population

From the EHR, we retrospectively collected clinical information from all adults (≥20 years old) admitted to the ED as inpatients (July 2016 to October 2016) at Chi-Mei Medical Center, a tertiary teaching hospital located in Southern Taiwan. Sepsis cases were assessed based on a manual chart review according to the Rhee clinical surveillance criteria [24]. Two experienced clinicians independently reviewed the medical records of the study cohorts, throughout the clinical course from ED arrival to hospital charge or death, to determine whether a patient had sepsis. Patients were excluded if they were (a) less than 20 years old, (b) identified as septic patients before ED admission. This study was reviewed and approved by the Institutional Review Board of Human Research at both the Chi-Mei Medical Centre and the Taoyuan General Hospital (IRB No: TYGH107014).

### 2.2. Sepsis Definitions

Sepsis was confirmed when either one of the following two conditions were fulfilled (Table 1): (1) the Sepsis-3 definition [1], that is, having a suspected infection (prescription of antibiotics and sampling of bodily fluids for microbiological culture) combined with evidence of organ dysfunction, defined by an increase in the Sequential Organ Failure Assessment (SOFA) score greater than or equal to two, and (2) having a suspected infection combined with evidence of hypoperfusion and shock, including lactate >2 mmol/L and the presence of vasopressor medications.

### 2.3. Predictor Variables

We collected the following clinical information which was available in the EHR: patient’s vitals upon arrival acquired by a triage nurses including; systolic blood pressure (SBP), diastolic blood pressure (DBP), respiratory rate (RR), Glasgow coma scale (GCS), body temperature (BT), heart rate (HR), and the first acquired laboratory study results during the patient’s stay at the emergency department, including complete blood count, lactate level, C-reactive protein (CRP), random glucose, sodium level (Na), potassium level (K), blood urea nitrogen (BUN), creatinine (Cr), glutamic oxaloacetic transaminase (GOT), glutamate pyruvate transaminase (GPT), total bilirubin (T.bil), high sensitivity Troponin I (hs-TnI), and creatine kinase-MB (CK-MB).

### 2.4. Model Development and Validation

The development dataset was split into training and testing sets (internal validation) with an 80–20 ratio in a stratified fashion to preserve the same prevalence of sepsis cases as in the development dataset. We developed eXtreme Gradient Boosting (XGBoost), a highly scalable end-to-end tree boosting system proposed by Chen and Guestrin [25], on the training cohort using all clinical variables and validated this model internally to stratify sepsis patients.

XGBoost does not require data normalisation of input features and has the ability to cope with sparse data. It surpasses traditional tree-based models by introducing regularisation to avoid overfitting, by utilising gradient boosting to ensemble multiple tree models for better performance, and by mitigating biases. The objective function was utilised in minimising logistic loss, and we used the grid search method to tune the hyper-parameters of our model. During the training process, five-fold cross validation was applied to reduce sample bias. We selected the threshold that gave the highest F2-score. The equation for calculating the F2-score is given below:

F2 = (5 × Precision × Recall)/(4 × Precision + Recall)

Modelling was developed using the software Python version 3.6.3 and XGBoost Package version 1.2.1.

### 2.5. Evaluating Model Performance

We present the performance of the XGBoost model on the internal validation data for identifying sepsis using AUC. We calculated accuracy, sensitivity, specificity, negative predictive value (NPV), and positive predictive value (PPV). We compared the performance of model with traditional clinical tools, namely SIRS and qSOFA.

### 2.6. Feature Selection

To determine the major predictors of stratifying sepsis patients, feature selection was performed. We used XGBoost’s built-in function in Python, “feature importances”, and analysed the top ranking features. This provided the information of the relative contribution of the corresponding feature to the model calculated by taking each feature’s contribution for each tree in the model. A higher ranked feature on the chart implies that it is more important for generating the prediction.

### 2.7. External Validation

A separate cohort of 1744 unique adult patients admitted to the ED at Taoyuan General Hospital, a regional hospital located in Northern Taiwan, from January 2018 to March 2018 was used for external validation. We collected the following information from external validation datasets: (1) patient’s underlying comorbidities; (2) if antibiotics were prescribed, the triage-to-drug time; (3) whether the discharge summary, either from the ED or after hospitalisation, contained sepsis-related diagnosis codes; (4) documented infection focus for the sepsis.

### 2.8. Statistical Analysis

Statistical analyses were performed for top ranking predictors of the model between the development dataset and the external validation dataset. Additionally, in the external validation dataset, with the prediction results of the machine learning model, we compared the comorbidities and the infection source for sepsis between the true positive group and the false negative group, and between the true negative and the false positive group. All of the statistical analyses were performed using SAS Enterprise Guide 8.3. Student’s *t*-test was used for continuous variables and the chi-squared test was used for categorical variables to evaluate differences between the two groups.

### 2.9. Promoting Interoperability

This article followed TRIPOD (Transparent Reporting of a Multivariable Prediction Model for Individual Prognosis or Diagnosis) guidelines [26] (Supplementary Table S1).

## 3. Results

### 3.1. Patient Characteristics

After applying the exclusion criteria, the final development cohort sizes were 8296 patients (6637 (80%) for training and 1659 (20%) for internal validation). However, the external validation cohort included 1744 patients, and 506 (29%) of them were sepsis patients. The mortality of sepsis patients in the development and the external validation dataset was 13.5% and 17%, respectively. In our external validation dataset, the average triage-to-antibiotic time for patients coded as having sepsis was 3.18 h, whereas the average triage-to-antibiotic time for true sepsis patients was 3.96 h (Table 2).

### 3.2. Model Performance for Identifying Sepsis Patients

**Figure 2:**
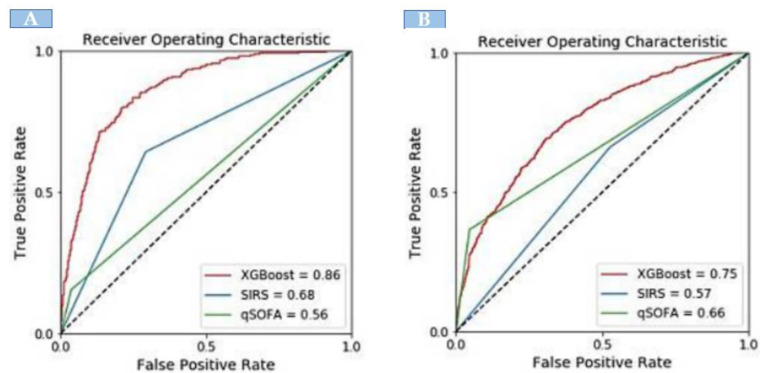
The area under the receiver operating curve comparison of XGBoost model with SIRS and qSOFA; (**A**) internal validation and (**B**) external validation.

When compared with the existing identification tools, the XGBoost model showed significantly greater discrimination of sepsis. In the development dataset, there were 1742 sepsis patients. The XGBoost model showed significantly greater discrimination (AUC: 0.86) in identifying sepsis patients. The area-under-the-curve (AUC) for external validation was 0.75. However, XGBoost exhibited a higher AUC compared with SIRS and qSOFA for identifying sepsis both in the internal and external validation sets (Figure 2).

Table 3 presents the performance comparison between the XGBoost model and traditional sepsis tools. In the internal validation, XGBoost had a sensitivity of 80% and specificity of 78%. For the identification of sepsis, SIRS had a sensitivity of 64% and specificity of 66%; qSOFA had a sensitivity of 35% and specificity of 96%. The predictive values of XGBoost (PPV: 0.47, NPV: 0.94) were higher than that of SIRS (PPV: 0.34, NPV: 0.77) and qSOFA (PPV: 0.76, NPV: 0.79). In the external validation, XGBoost had a sensitivity of 67% and specificity of 70%, which was higher than SIRS (sensitivity: 66%, specificity: 47%), and qSOFA (sensitivity: 36%, specificity: 89%). The PPV and NPV for XGBoost were 48% and 84%, respectively.

### 3.3. Most Important Predictors of Sepsis as Assessed with the XGBoost Model

**Figure 3:**
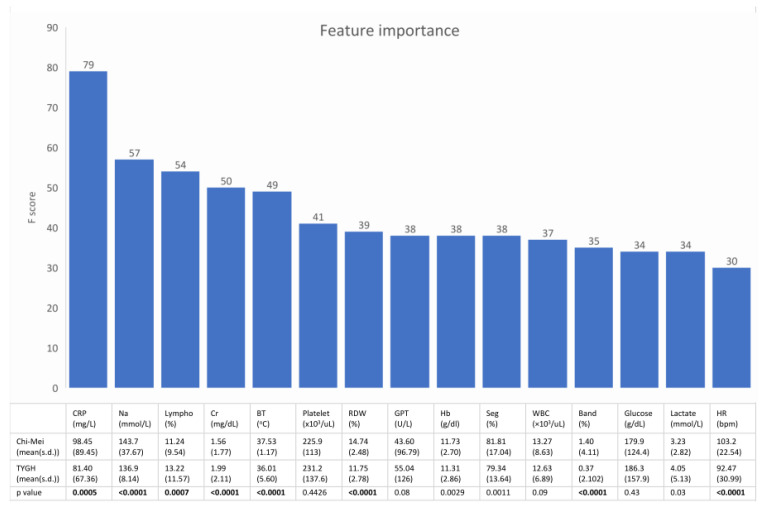
The upper bar plot shows the 15 top ranking features of the machine learning model. The lower table is the statistical analysis of these features between septic patients in the development dataset and septic patients in the external validation dataset. Most of the important predictors had a heterogeneous distribution between the two datasets. (Lympho = percentage of lymphocytes in the differential of the complete blood count).

Figure 3 shows the feature rankings of the XGBoost model and the statistical analysis of the 15 top-ranking features between septic patients in the development dataset and septic patients in the external validation dataset. These 15 top-ranking features accounted for 65% of the total weight.

### 3.4. Potential Clinical Confounders of Model Performance

Table 4 shows the statistical analysis of comorbidities and infection source for sepsis between the true positive (TP) group and the false negative (FN) group, and between the true negative (TN) and the false positive (FP) group in the external validation dataset after implementing the machine learning model. The result suggests that age, presence of coronary artery disease, chronic kidney disease, urinary tract infection, and pneumonia might interfere with the model performance.

## 4. Discussion

### 4.1. Main Findings

In the present study, we developed and externally validated an ML model to correctly identify sepsis in patients admitted to the ED. The XGBoost model demonstrated great performance with an AUROC of 0.85 and 0.75 in the internal and external validation, respectively. Our current model significantly outperformed the other clinically available stratifying tools. The findings of our study suggest that the XGBoost model has an important clinical role in identifying sepsis patients in the ED.

### 4.2. The Pivotal Role of the ED in Developing a Sepsis Identification Model

International consensus has continued to emphasise the benefit of the early recognition of sepsis, followed by timely treatment on patient outcomes [27]. Because the ED is generally the initial arrival site for septic patients [24,28], this recommendation underscores the pivotal role of optimising sepsis identification in the ED.

In order to minimise the risk of bias, our study followed the published recommendations [29,30] to develop and externally validate an ML model for sepsis identification in the ED. The study shows that ML, even when externally validated in a discrepant dataset, could demonstrate acceptable discriminative power in identifying sepsis patients and outperformed the existing SIRS and qSOFA criteria.

In contrast to the neural network, which is like a “black box”, we adopted the XGBoost model for its better clinical interpretability. According to Figure 2, the top-ranking features of the machine learning model, such as CRP, Na, Cr, BP, and platelets, correspond well with the key clinical features that physicians use to identify sepsis or to assess the severity of sepsis.

### 4.3. Machine Learning Might Help Shorten the Triage-to-Antibiotic Time

Early administration of antibiotics is crucial for improving outcomes in septic patients. A delay in starting antibiotics is associated with increased in-hospital mortality [30,31], especially in patients with septic shock. The survival rate can drop by 7.6% with each hour of delay after hypotension has developed [30]. However, the door-to-antibiotic time varies significantly among different attending physicians in the ED setting [32]; studies have shown that the median interval from the time of presentation to antibiotic administration can range from 71 to 359 min, with a median of 4 h [33,34]. Physician and hospital related factors that contribute to such variation in time to antibiotic treatment include diagnostic delays, computerised order entry systems and ED crowding [32]. In our external validation dataset, the average triage-to-antibiotic time for patients coded as having sepsis was 3.18 h, whereas the average triage-to-antibiotic time for true sepsis patients was 3.96 h. Previous prospective interventional studies have demonstrated that the implementation of computerised systems for assisting sepsis identification could shorten the time to intervention for patients who triggered the sepsis alert [35,36,37]. These results support the potential of ML for facilitating timely sepsis care and improving patient outcomes.

### 4.4. Factors Associated with the Heterogeneity of Model Performance

Variations in the predictive performance of a model across different patient cohorts have been well-documented for models developed using traditional statistical approaches [38,39,40,41]. However, the impact of heterogeneity in patient characteristics on model performance has rarely been assessed for sepsis prediction models in the previous literature [42,43,44,45]. Among published models targeting sepsis identification in the ED setting, only the one developed by Faisal et al. [46] was examined by external validation, which also showed discrepant performance between the development dataset and the external validation dataset. However, the authors did not elucidate the reason for the discrepancy. Heterogeneity between the two independent datasets can generally be categorised into “figure drifting” (i.e., differences in predictors) and “label drifting” (i.e., differences in outcomes) [45]. Figure drifting and label drifting can originate from the data itself (e.g., differences in prevalence or severity), or the criteria used for determining the predictor values or outcomes [41,47]. Different designs of external validation, such as temporal (i.e., same institute, different study period), institutional (i.e., two geographically adjacent institutes), and geographical (i.e., two institutes in different regions, or even countries), determine the extent of heterogeneity between the external validation dataset and the source dataset.

In the external validation dataset, we reaffirmed that ICD coding may not be an appropriate surrogate for the Sepsis-3 criteria as the reference standard to identify sepsis [27,48,49]. However, even when the determination of outcomes (i.e., presence of sepsis) was controlled by a standardised review process in this study, our results still show that the performance of the ML model declines when the prevalence of the outcome and the distribution of predictors differed between the two geographically and temporally independent datasets. Patient comorbidities and infection sources, although not predictors, also appeared to interfere with model performance. This finding indicates that the intended population should be specifically defined to ensure the clinical applicability of the model. Correspondingly, a thorough examination of data heterogeneity should be conducted to judge the efficacy of ML on the targeted clinical setting.

### 4.5. Study Limitations

There are several limitations of this study. First, the development dataset and validation dataset were both derived from a single institute, which suggested that selection bias might exists. However, the in-hospital mortality for patients with sepsis present on admission, using the Sepsis-3 criteria for sepsis identification was similar to the previous epidemiologic result (13.5% in our study vs. 13.4% in previous study) [24]. Second, heterogeneity in study designs has been shown to hinder the comparison of performance among different models [18,27]. Therefore, we could hardly compare the performance of our model with previous models [50,51,52,53] because considerable heterogeneity was found among our study and previous studies, including the patient characteristics (e.g., all ED visits versus only ED admissions, with differences in sepsis prevalence), selecting predictors (e.g., vitals, lab test results versus text data from the EHR), the reference standard for sepsis (ICD coding versus the Sepsis-3 criteria), and diagnostic outcomes (e.g., sepsis versus severe sepsis and septic shock versus mortality). Third, information on patient age, comorbidities, and infectious focus for sepsis was lacking in the development dataset for the comparison of the two datasets. However, the collected vital signs and lab tests results appeared to be sufficient to reflect the discrepancies in clinical manifestations between the two patient cohorts. Finally, because our study results suggested several patient characteristics (such as age, certain comorbidities, and infection focus) might interfere with the model performance, whether the implementation of a ML model can shorten the triage-to-antibiotics time requires future, prospective interventional trials.

## 5. Conclusions

Using commonly available clinical variables, we developed and externally validated a ML model to effectively identify sepsis patients in the ED. This study demonstrated that the XGBoost model outperformed the pre-existing conventional tools in identifying sepsis patients; however, we also revealed that differences in patient characteristics, while being key predictors, could reduce model performance. This finding reinforces the recommendation of performing external validation to ensure the generalisability of clinical decision support models. Because heterogeneity among patient cohorts seems inevitable, future studies are needed to solve this model adaptation problem.

## Figures and Tables

**Table 1:** Sepsis-3 (revised) definition for sepsis compared with the traditional sepsis definition.

	Traditional Definition	Sepsis-3 Definition
Sepsis	Suspicious/known infection + ≥2 SIRS	Suspicious/known infection + rise in SOFA score ≥2
Severe sepsis	Sepsis + SBP < 90 mmHg or MAP < 65 mmHg, lactate > 2 mmol/L (18 mg/dL) INR > 1.5 or a PTT > 60 s Bilirubin > 34 μmol/L Urine output < 0.5 mL/kg/h for 2 h Creatinine > 177 μmol/L Platelets < 100 × 10^9^/L SpO_2_ < 90%on room air	Not a category
Septic Shock	Sepsis + hypotension after adequate fluid resuscitation	Sepsis + Vasopressors needed for MAP >65 mmHg + Lactate >2 mmol/L after adequate fluid resuscitation

**Table 2:** Description of the development dataset and the external validation dataset.

	Development Dataset		Validation Dataset	
**Case number**	8296		1744	
**Geographical region**	Southern Taiwan		Northern Taiwan	
**Data collection period**	1 July 2016 to 31 October 2016		1 January 2018 to 31 March 2018	
**Study design**	Retrospective		Retrospective	
**Setting**	A tertiary teaching hospital		A regional hospital	
**Inclusion criteria**	All the adult ED visits (≥20 years old) admitted as inpatient without further transferring during the whole hospitalisation			
**Reference standard for sepsis**	Sepsis-3 definition			
**Prevalence of sepsis**	21%		29%	
**Mortality for sepsis**	13.5%		17%	
**Mean** **length of stay (days)**	9.8		6.3	
**Model predictors**	mean	s.d.	mean	s.d.
Average of SIRS	1.22	1.02	1.71	1.04
Average of qSOFA	0.33	0.62	0.57	0.76
**Vital signs at triage**				
SBP	142.2	33.21	132.5	36.94
DBP	84.57	17.94	76.52	23.62
RR	18.24	3.48	20.30	3.83
GCS	14.27	2.23	13.65	2.99
BT	36.93	0.96	36.46	3.42
HR	92.80	21.74	93.44	25.54
**Initial lab results at ED**				
WBC	10.94	6.14	11.45	5.74
Segment	76.30	15.77	76.84	13.07
Band	0.45	2.28	0.15	1.26
Eosinophil	1.38	2.38	1.21	1.95
Basophil	0.34	0.36	0.34	0.41
Lymphocyte	16.45	11.14	15.32	11.07
Platelet	232.6	96.85	247.6	121.9
Haemoglobin	12.52	2.87	12.04	2.91
Haematocrit	36.77	7.53	37.01	8.38
MCH	29.45	3.3	29.24	3.46
MCHC	33.16	1.64	32.34	1.74
MCV	88.76	8.45	90.25	8.87
RBC	4.16	0.90	4.135	1.00
RDW	14.24	2.39	11.29	2.34
Lactate	3.02	2.80	3.15	4.01
CRP	60.08	74.65	67.66	66.87
Glucose	159.2	104.2	171.6	130.8
Na	142.3	29.38	137	6.50
K	4.08	1.37	4.14	0.73
BUN	30.17	27.10	29.80	27.36
Cr	1.39	1.80	1.67	2.07
GOT	87.29	187	61.68	225.7
GPT	42.1	114.2	42.91	104.4
T.bil	2.95	4.98	1.43	1.91
hsTnI	1043.8	21569.8	811.8	20073.5
CK-MB	8.24	32.53	6.10	22.46

Note: MCH = mean corpuscular haemoglobin; MCHC = mean corpuscular haemoglobin concentration; MCV = mean corpuscular volume; RBC = red blood cell; RDW= red cell distribution width; SBP = systolic blood pressure; DBP = diastolic blood pressure; RR = respiratory rate; GCS = Glasgow Coma Scale; BT = blood temperature; HR = heart rate; CRP = C-reactive protein; Na = sodium; K = potassium; BUN = blood urea nitrogen; Cr = creatinine; GOT= aspartate aminotransferase; T.bil = bilirubin test; hsTnI = high sensitivity troponin; CK-MB = creatine kinase-MB.

**Table 3:** Diagnostic performance of the identification of sepsis.

Model Performance	XGBoost	SIRS	qSOFA
**Internal validation**			
Accuracy	0.78	0.69	0.79
Sensitivity	0.80	0.64	0.35
Specificity	0.78	0.66	0.96
PPV	0.47	0.37	0.53
NPV	0.94	0.88	0.81
**External validation**			
Accuracy	0.70	0.34	0.75
Sensitivity	0.67	0.66	0.36
Specificity	0.70	0.47	0.89
PPV	0.48	0.34	0.76
NPV	0.84	0.77	0.79

Note: PPV = positive predictive value, NPV = negative predictive value.

**Table 4:** Statistical analysis of comorbidities and infection source among patients in the external validation dataset, divided according to the model output.

	TP	FN	*p*-Value	TN	FP	*p*-Value
**Age (years)**	74.4	67.8	<0.001	61.8	59.9	0.1063
**Presence of comorbidity (%)**						
Diabetes mellitus	40.18	36.36	0.4097	27.75	29.23	0.5967
Hypertension	49.56	49.09	0.9212	41.63	36.89	0.1204
Coronary artery disease	14.37	13.33	0.7531	15.6	9.02	0.0021
Chronic kidney disease	12.02	9.7	0.4378	8.6	5.19	0.0388
End-stage renal disease	7.62	5.45	0.3671	4.93	5.74	0.5586
Cerebrovascular accident	19.35	16.36	0.4154	5.05	7.38	0.1074
Congestive heart failure	9.09	7.27	0.4917	5.16	3.55	0.2216
Malignancy	11.14	10.91	0.9317	8.37	11.2	0.116
**Presence of infection focus (%)**						
Urinary tract infection	28.45	20.61	0.0591	6.65	19.13	<0.0001
Cellulitis	3.52	3.03	0.7749	6.77	7.1	0.8302
Pneumonia	24.05	8.48	<0.0001	4.93	24.32	<0.0001
Intra-abdominal infection	2.93	6.06	0.0905	6.31	6.28	0.9878

Note: TP: True positive; FN: False negative; TN: True negative; FP: False positive.

## Supplementary Materials

The following are available online at https://www.mdpi.com/article/10.3390/jpm11111055/s1, Table S1: TRIPOD checklist.
